# Diagnostic Value of EBUS-TBNA for Lung Cancer with Non-Enlarged Lymph Nodes: A Study in a Tuberculosis-Endemic Country

**DOI:** 10.1371/journal.pone.0016877

**Published:** 2011-02-25

**Authors:** Chih-Hsi Kuo, Hao-Cheng Chen, Fu-Tsai Chung, Yu-Lun Lo, Kang-Yun Lee, Chih-Wei Wang, Wen-Han Kuo, Tzu-Chen Yen, Han-Pin Kuo

**Affiliations:** 1 Department of Thoracic Medicine, Chang Gung Memorial Hospital, Chang Gung University School of Medicine, Taipei, Taiwan; 2 Department of Pathology, Chang Gung Memorial Hospital, Taipei, Taiwan; 3 Department of Nuclear Medicine and Molecular Imaging Center, Chang Gung Memorial Hospital, Taipei, Taiwan; University of Texas, M.D. Anderson Cancer Center, United States of America

## Abstract

**Background:**

In tuberculosis (TB)-endemic areas, contrast-enhanced computed tomography (CT) and positron emission tomography (PET) findings of lung cancer patients with non-enlarged lymph nodes are frequently discrepant. Endobronchial ultrasound-guided transbronchial aspiration (EBUS-TBNA) enables real-time nodal sampling, and thereby improves nodal diagnosis accuracy. This study aimed to compare the accuracy of nodal diagnosis by using EBUS-TBNA, and PET.

**Methods:**

We studied 43 lung cancer patients with CT-defined non-enlarged mediastinal and hilar lymph nodes and examined 78 lymph nodes using EBUS-TBNA.

**Results:**

The sensitivity, specificity, positive predictive value, and negative predictive value of EBUS-TBNA were 80.6%, 100%, 100%, and 85.7%, respectively. PET had low specificity (18.9%) and a low positive predictive value (44.4%). The diagnostic accuracy of EBUS-TBNA was higher than that of PET (91% vs. 47.4%; *p*<0.001). Compared to CT-based nodal assessment, PET yielded a positive diagnostic impact in 36.9% nodes, a negative diagnostic impact in 46.2% nodes, and no diagnostic impact in 16.9% nodes. Patients with lymph nodes showing negative PET diagnostic impact had a high incidence of previous pulmonary TB. Multivariate analysis indicated that detection of hilar nodes on PET was an independent predictor of negative diagnostic impact of PET.

**Conclusion:**

In a TB-endemic area with a condition of CT-defined non-enlarged lymph node, the negative diagnostic impact of PET limits its clinical usefulness for nodal staging; therefore, EBUS-TBNA, which facilitates direct diagnosis, is preferred.

## Introduction

Lung cancer remains a fatal disease worldwide, and surgical treatment may offer long-term survival. However, the indication and outcome of surgical resection depend on accurate preoperative staging of the cancer and the extent of intraoperative lymph node dissection [1], [2], [3]. Therefore, accurate lymph node staging in patients with non-small cell lung cancer (NSCLC) is crucial for planning the optimal treatment. Conventional contrast-enhanced computed tomography (CT) has been used to identify enlarged lymph nodes; lymph nodes >1 cm are defined as nodal metastatic lesions. However, because it is moderately sensitive and pecific, contrast-enhanced CT carries a substantial risk of understaging small nodal metastasis and overstaging inflammatory lymphadenitis [4].

Positron emission tomography (PET) using fluorine-18 fluorodeoxyglucose (^18^F-FDG) provides functional information about tumor metabolism and has been used as a non-invasive alternative to contrast-enhanced CT for nodal staging in NSCLC [5], [6], [7]. In some countries, FDG-PET is increasingly being used for lymph node staging in NSCLC when lymph node enlargement is not detected by CT. Therefore, the accuracy afforded by FDG-PET may substantially alter the treatment strategy in institutions in which mediastinoscopy is unavailable for lymph node sampling. However, abnormal FDG uptake is believed to frequently occur in granulomatous and inflammatory diseases [8], [9]. In tuberculosis (TB)-endemic areas such as Eastern Asia, FDG-PET has been reported to have low specificity and a positive predictive value in nodal staging in NSCLC [10], [11], [12]. Therefore, in such areas, the PET scan does not appear to replace mediastinoscopy for nodal staging of NSCLC [11], especially in the case of potentially operable patients without enlarged mediastinal lymph nodes.

The recently developed endobronchial ultrasound-guided transbronchial aspiration (EBUS-TBNA) procedure enables direct and real-time aspiration of mediastinal and hilar lymph nodes and is a less invasive alternative to mediastinoscopy for nodal staging [13], [14]. Unlike CT and FDG-PET, in which lymph node stage is determined on the basis of the results of image analysis, EBUS-TBNA enables direct nodal sampling; therefore, nodal staging can be performed on the basis of the results of cytological or pathological examination. However, EBUS-TBNA has been reported to have variable sensitivity and negative predictive value [13], [15], especially in cases wherein the size of the lymph nodes has been reduced by induction chemotherapy [16]. However, EBUS-TBNA has been reported to show high sensitivity and specificity for detecting small nodal metastasis in NSCLC patients without significant mediastinal lymph node enlargement on CT [17], [18]. Therefore, whether EBUS-TBNA shows high diagnostic accuracy for nodal staging in lung cancer patients without enlarged mediastinal lymph nodes on CT in a TB-endemic country—a condition for which FDG-PET has shown much negative diagnostic impact, i.e. false-positive results—remains to be clarified.

In this study, we primarily aimed to compare the diagnostic accuracy of EBUS-TBNA and FDG-PET in lung cancer patients with CT-defined non-enlarged mediastinal and hilar lymph nodes. Further, we also aimed to look at the diagnostic impact of FDG-PET in this condition, and investigated the characteristics of lymph nodes which accounted for either negative or positive diagnostic impact on FDG-PET.

## Materials and Methods

### Study subjects

Between January 2007 and January 2009, nodal sampling by using EBUS-TBNA was performed in 43 lung cancer patients with non-enlarged mediastinal and hilar lymph nodes defined by contrast-enhanced CT examination. Written informed consent was obtained from all study subjects and the institutional review board of Chang Gung Memorial Hospital approved the study (No. 98-3639A3).

### Imaging techniques

Contrast-enhanced CT was initially performed in the 45 patients included in the study to examine lung parenchymal and mediastinal lesions. CT images were obtained from the thoracic inlet to the adrenal gland, and the images were assessed by an experienced radiologist who was blinded to the FDG-PET and pathology reports. Non-enlarged mediastinal and hilar lymph nodes were defined as lymph nodes with short-axis diameters ≤10 mm on contrast-enhanced CT of the chest. The attending physician referred patients for FDG-PET by considering each patient's will and payment of personal medical insurance. Each patient was instructed to fast for 6 h before undergoing PET and was intravenously injected with 370 MBq (10 mCi) of ^18^F-FDG 60 min before the examination. ^18^F-FDG PET images were obtained using an ECAT EXACT HR+ camera (Siemens/CTI Inc., Knoxville, TN) by using full width at half-maximum of 4.5 mm and a transaxial field of view of 15 cm. Transaxial, sagittal, and coronal tomographs were assessed primarily to determine obvious abnormal foci of increased FDG uptake. All the images were double-read by two nuclear physicians with more than 6 years experience in PET interpretation, and the discrepancies were resolved by discussion. A visual analysis score (VAS) using a 4-point scale was primarily used to determine ^18^F-FDG uptake in the lymph nodes, and the standardized uptake value was used as an accessory reference. The 4-point scale considered both the intensity of ^18^F-FDG uptake in the lymph nodes and the contrast between the background mediastinum and lymph nodes showing ^18^F-FDG accumulation. Accordingly, the 4-point scale was developed as follows: 1, intensity of focal uptake of ^18^F-FDG lower than that of mediastinal uptake (benign lesion); 2, intensity of focal uptake of ^18^F-FDG equal to that of mediastinal uptake (equivocal for malignancy); 3, intensity of focal uptake of ^18^F-FDG greater than that of mediastinal uptake (possibility of malignancy); and 4, intensity of focal uptake of ^18^F-FDG significantly greater than that of mediastinal uptake (high probability of malignancy). A VAS of 3 or 4 was considered positive, whereas a score of 1 or 2 was considered negative. PET scan results of 32 patients were available at the time of data analysis. Two patients were excluded from EBUS-TBNA test due to metastatic disease noted by PET. Compared to CT-based nodal assessment, we interpreted a true-positive PET result as having positive diagnostic impact and a false-positive PET result as having negative diagnostic impact, categorizing either a true-negative or false-negative PET result as having no diagnostic impact (i.e. PET showed a same nodal assessment as CT).

### EBUS-TBNA

All of the patients underwent TBNA via a flexible ultrasound-guided bronchoscope with a linear scanning probe on the tip (BF-UC206F-OL8, Olympus) EBUS-TBNA were performed by two well-trained operators both have had experience in more than 30 cases, and the bronchoscopic operators were blinded to the PET scan result. The curved probe scanned in a direction parallel to that of the bronchoscope insertion, and the obtained images were linked to an ultrasound scanner (EU-2000C, Olympus) with Doppler-flow imaging. Conscious sedation was induced in all patients by using midazolam before the bronchoscope was inserted. Ultrasound examination of the hilar lymph node (stations 10, 11) was followed by subcarinal (station 7) and mediastinal lymph nodes (stations 4, 2, 1). When a lymph node was visualized, the picture was recorded and selected cursors were used to measure its size 2-dimensionally. Lymph nodes with short-axis diameters >5 mm were selected for subsequent real-time EBUS-guided TBNA with a 22-gauge needle (NA-201SX-4022, Olympus). The needle was passed at least 2 times for each targeted lymph node. An internal sheath was placed inside the needle to avoid contamination of the bronchial epithelium during each puncture, and the sheath was removed after passage of the needle into the targeted lymph node. Cytological examination of the specimens was performed by a pathologist blinded to the patients' clinical histories and imaging results. Pathological examination of the specimen was also performed when a tissue core was obtained by TBNA.

### Statistical analysis

The result of each diagnostic modality was compared with that of the surgical specimen obtained by extensive lymph node dissection in 30 (69.8%) patients. Mediastinoscopy was performed to approach N2 level lymph nodes in 5 patients; in them, biopsy specimens were used for standard comparison. Eight patients refused any sort of approach for diagnosis of mediastinal lymph node involvement or treatment. Neither did they receive surgery nor chemotherapy or radiotherapy. A series of CT scan image follow-up was done in those patients; in that, 20% increase in short-axis of a lymph node within 3 months was used to defined pre-existing metastasis The sensitivity, specificity, positive predictive value, and negative predictive value of each diagnostic modality were calculated according to the standard definition. The chi-squared test was used to compare differences between groups. All reported *p* values were two-sided, and a *p* value <0.05 was considered statistically significant.

## Results

### Patients and lymph node characteristics

We included a total of 43 lung cancer patients (25 men and 18 women; median age: 66 years) with non-enlarged mediastinal and hilar lymph nodes. Baseline characteristics of the patients are shown in Table 1. Results are expressed as mean (SD). The patients were diagnosed with the following conditions: adenocarcinoma in 29 (69%), squamous cell carcinoma in 4 (9.5%), large cell carcinoma in 1 (2.4%), undetermined NSCLC in 6 (14.3%), and small cell carcinoma in 2 (4.8%). We performed EBUS-TBNA in these patients and examined a total of 78 lymph nodes that had a mean long axis and mean short axis of 9.5 mm (3.8 mm) and 6.8 mm (1.4 mm), respectively. Lymph nodes located in the following regions were targeted: highest mediastinal, 1 (1.3%); right upper paratracheal, 3 (3.8%); left upper paratracheal, 1 (1.3%); right lower paratracheal, 20 (47.6%); left lower paratracheal, 9 (21.4%); subcarinal, 23 (54.8%); right hilar, 12 (28.6%); left hilar, 5 (11.9%); right interlobar, 2 (2.6%); and left interlobar, 2 (2.6%).

**Table 1:** Baseline characteristics of lung cancer patients with CT-defined radiologically normal mediastinum.

Variables	
**Total patients, no. (men/women)**	43 (25/18)
**Median age, years**	66
**Lymph node size by CT, mm, mean (SD)**	
Short axis	6.8 (1.4)
**Final diagnosis, no. (%)**	
Adenocarcinoma	29 (69)
Squamous cell carcinoma	4 (9.5)
Large cell carcinoma	1 (2.4)
Undetermined NSCLC	6 (14.3)
Small cell carcinoma	2 (4.8)
**Location of lymph nodes targeted by EBUS-TBNA, no. (%)**	
Highest mediastinal (#1)	1 (1.3)
Right upper paratracheal (#2R)	3 (3.8)
Left upper paratracheal (#2L)	1 (1.3)
Right lower paratracheal (#4R)	20 (47.6)
Left lower paratracheal (#4L)	9 (21.4)
Subcarinal (#7)	23 (54.8)
Right hilar (#10R)	12 (28.6)
Left hilar (#10L)	5 (11.9)
Right interlobar (#11R)	2 (2.6)
Left interlobar (#11L)	2 (2.6)
Total	78 (100)

CT: computed tomography; NSCLC: non-small cell lung cancer; EBUS-TBNA: endobronchial ultrasound-guided transbronchial aspiration.

### Diagnostic value of PET and EBUS-TBNA

**Figure 1:**
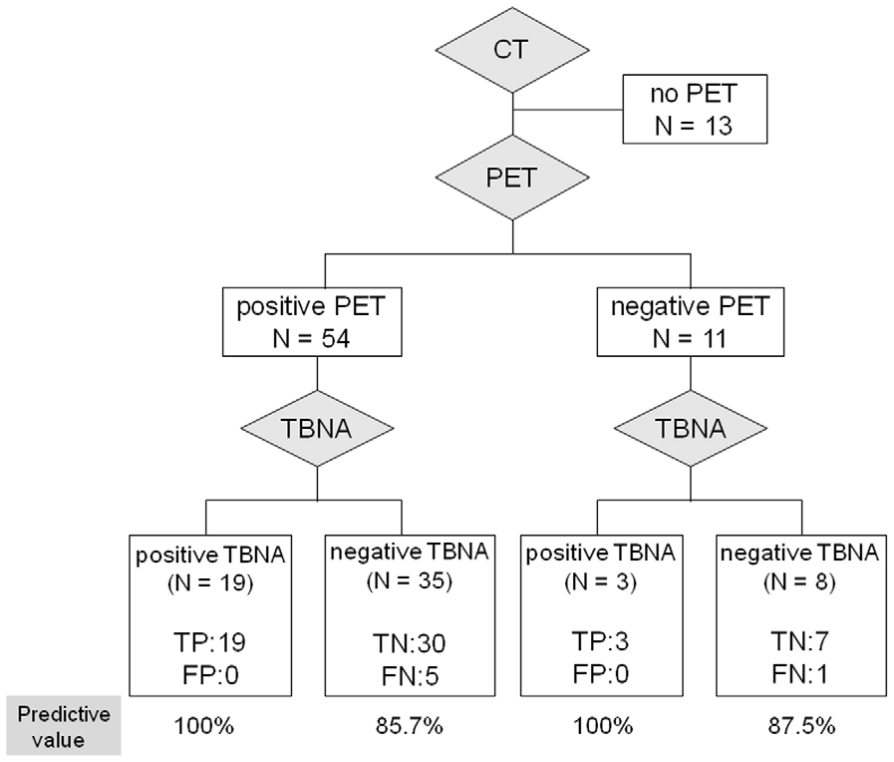
Predictive value of EBUS-TBNA stratified by PET scan results. TP, true-positive; TN, true-negative; FP, false-positive; FN, false-negative.

The results of each diagnostic modality on a per lymph node basis are shown in Table 2. The sensitivity, specificity, positive predictive value, and negative predictive value were 80.6%, 100%, 100%, and 85.7%, for EBUS-TBNA and 85.7%, 18.9%, 44.4%, and 63.6% for PET, respectively. Thus, in our study, the diagnostic accuracy of EBUS-TBNA was higher than that of PET (91% vs. 47.4%; *p*<0.001). Further, when primary tumor site was considered during interpretation of the PET results, the contralateral lymph nodes with ^18^F-FDG uptake scores of 3 or 4 were deemed PET negative. Thus, the accuracy of the modified method in which PET results were interpreted on the basis of the primary tumor site was still lower than that of the EBUS-TBNA results (52.3% vs. 91%, *p*<0.001). When lymph nodes were positive for malignancy by EBUS-TBNA irrespective of PET findings (PET-positive, n = 19; PET-negative, n = 3), the predictive value of EBUS-TBNA for lymph nodes diagnosis was 100% (Fig. 1). When lymph nodes were negative for malignancy by both EBUS-TBNA and PET (n = 8), the predictive value of both tests were 87.5%. In the case of EBUS-TBNA-negative and PET-positive lymph nodes (n = 35), predictive value was 85.7% in favor of the negative EBUS-TBNA result.

**Table 2:** Diagnostic values of PET and EBUS-TBNA in lung cancer patients with CT-defined radiologically normal mediastinum.

Tests	Sensitivity (%)	Specificity (%)	PPV (%)	NPV (%)	Accuracy (%)
PET	85.7	18.9	44.4	63.6	47.7
PET*	69.2	41.0	43.9	66.7	52.3
EBUS-TBNA	80.6	100	100	85.7	91.0

*PET judgment directed by primary tumor location: Contralateral lymph nodes with 3 or 4-point scale FDG uptake were judged as having a negative PET result. CT: computed tomography; PET: positron emission tomography; EBUS-TBNA: endobronchial ultrasound-guided transbronchial aspiration.

### Diagnostic impact of PET

**Figure 2:**
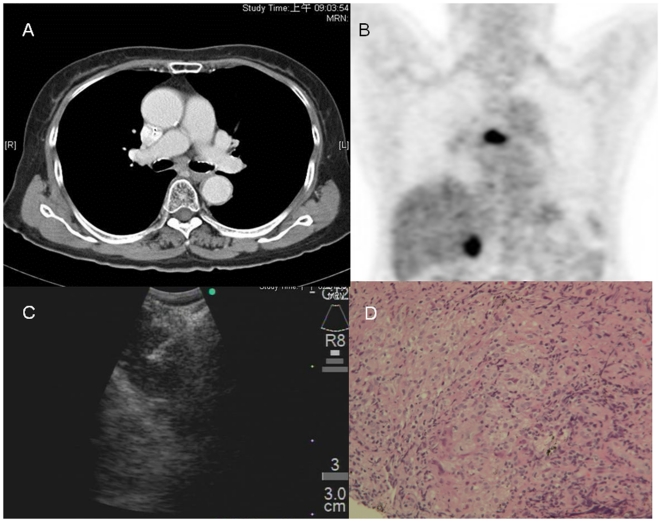
A representative case of a PET scan with negative impact. (A) The contrast-enhanced CT scan shows small lymph nodes measuring 0.8×0.6 cm in the subcarinal area. (B) The PET scan shows increased FDG uptake at the corresponding site (gall bladder uptake due to cholecystitis). (C) The subcarinal lymph node was later targeted by EBUS-TBNA(D) A tissue core, which revealed granulomatous inflammation consisting of epithelioid histiocytes (upper right; H&E stain, 200×) was obtained.

**Figure 3:**
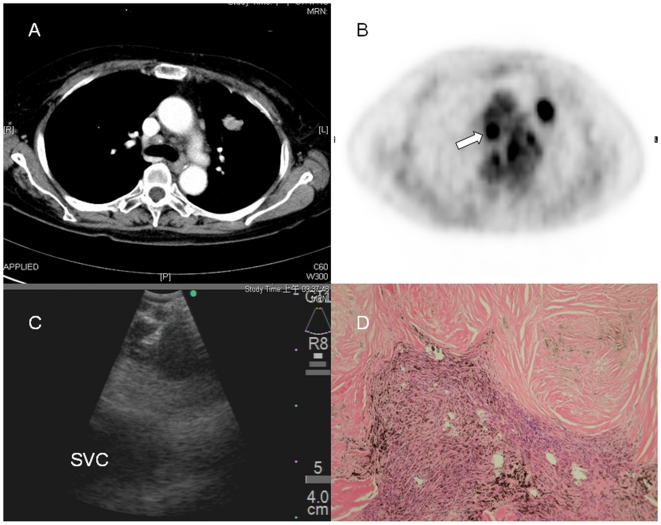
A representative case of a PET scan with negative impact. (A) The contrast-enhanced CT scan shows lymph node measuring 0.8×0.8 cm in para-tracheal area (4R). (B) The PET scan shows strong FDG uptake (arrow) at the corresponding site. (C) Lymph node is targeted by EBUS-TBNA, with the superior vena cava (SVC) lies beneath. (D) The pathology reveals collagenized nodules and anthracotic pigment-laden macrophages (H&E stain, 100X). Silica particles were identified under polarized light (not shown).

Compared to CT-based nodal assessment, 24 nodes detected by PET were true-positive, yielding a 36.9% positive impact incidence (Table 3). Thirty nodes detected by PET were false-positive, which accounted for a 46.2% negative impact incidence. Seven and 4 lymph nodes were PET-true-negative and PET-false-negative, respectively, and were together responsible for a 16.9% incidence of no impact. Patients with lymph nodes yielding negative PET scan diagnostic impact were thoroughly investigated to rule out the presence of pulmonary diseases other than lung cancer. Of the 17 patients examined, 7 (41.2%) had previous pulmonary TB, 3 (17.6%) had pneumoconiosis, 1 (5.9%) had pulmonary fibrosis, and 6 (35.3%) had no history of pulmonary disease. Rrepresentative cases of PET-negative diagnostic impact due to previous pulmonary TB with granulomatous nodal inflammation and anthracosilicosis diagnosed by EBUS-TBNAare shown in Fig. 2 and 3.

**Table 3:** Diagnostic impact of PET in CT-defined radiologically normal mediastinum.

Impact	Positive	None		Negative
Diagnosis	TP	TN	FN	FP*
No. (%)	24 (36.9%)	7 (10.8%)	4 (6.1%)	30 (46.2%)

*Seven patients (41.2%) previously had pulmonary tuberculosis, 3 (17.6%) previously had pneumoconiosis, 1 (5.9%) previously had pulmonary fibrosis, and 6 (35.5%) had no previous lung disease.

### Factors predicting the diagnostic impact of PET

The characteristics of lymph nodes yielding PET-positive impact were compared to those yielding PET-negative impact (Table 4). Four variables, namely lymph node size, lymph node location, PET scale, and presence of calcification were reviewed as factors predicting the diagnostic impact of PET. Among the PET-positive impact lymph nodes, 21 (87.5%) were located in the mediastinum and 3 (12.5%) were located in the hilum. In contrast, in the PET-negative impact group, 14 (46.7%) lymph nodes were located in the mediastinum and 16 (53.3%) were located in the hilum. In the PET-positive impact groups, 7 (29.2%) lymph nodes were scored as 3 and 17 (70.8%) were scored as 4. In contrast, in the PET-negative impact group, 20 (66.7%) lymph nodes were scored as 3 and 10 (33.3%) were scored as 4. Therefore, PET-positive hilar lymph nodes (53.3% vs. 12.5%, *p*<0.01) and lymph nodes scored as 3 (66.7% vs. 29.2%, *p*<0.05) were considered significant predictors of PET-negative nodal diagnostic impact. However, lymph node size and the presence of lymph node calcification did not predict PET result accuracy. The contribution of each predictor yielding PET-negative diagnostic impact was analyzed by multivariate logistic regression. The PET-positive finding of hilar lymph nodes (odds ratio [OR]: 5.75, 95% CI: 1.32–25.11, *p*<0.05), not lymph nodes scored as 3 points (OR: 3.21, 95% CI: 0.86–12.05, *p* = 0.08), was identified as the single independent predictor for PET-negative diagnostic impact (Table 5).

**Table 4:** Predictors for nodal diagnostic impact of PET scan in CT-defined radiologically normal mediastinum.

Variables	Positive impact	Negative impact	*p* value
**Size**			
short axis, mm, mean (SD)	6.9 (1.5)	6.5 (1.1)	0.35
**Location**			
hilar (#10, #11)	3	16	
mediastinal (#1, #2, #4, #7)	21	14	0.003
**PET scale**			
3-points	7	20	
4-points	17	20	0.01
**Calcification**			
with	3	7	
without	21	23	0.48

**Table 5:** Multivariate logistic regression analysis of predictors for negative nodal diagnostic impact of PET scan.

Variables	OR (95% CI)	*p* value
**Size**		
short axis	0.97 (0.59–1.60)	0.90
**Location**		
hilar vs. mediastinal	5.75 (1.32–25.11)	0.01
**PET scale**		
3 points vs. 4-points	3.21 (0.86–12.05)	0.08
**Calcification**		
with vs. without	1.09 (0.20–6.00)	0.93

### Diagnostic value of PET in determining distant metastasis

In 32 patients receiving PET exam, 3 were suspicious of distant metastasis. Two of them were excluded from EBUS-TBNA study due to lumbar spine metastasis proved by pathology, and the other one was proved to have cholecystitis. Hence; in the present study, the sensitivity, specificity and accuracy for PET to identified distant metastasis were 100%, 96.7% and 96.9%, respectively.

## Discussion

In our study, EBUS-TBNA had higher accuracy than PET for nodal diagnosis of lung cancer with CT-defined non-enlarged lymph nodes. In addition, presence of an ^18^F-FDG-positive hilar lymph node was an independent predictor for PET-negative diagnostic impact. Moreover, patients with lymph nodes yielding a PET-negative diagnostic impact showed a high incidence of inflammatory lung disease, especially those with previous pulmonary TB.

The strength and limitation of EBUS-TBNA in diagnosing lymph nodes metastasis has been reported in either TB or non-TB endemic country. The specificity was 100% in general; with a wide range of sensitivity from 69% to 94% [14], [15], [17], [19], [20], [21]. However, the diagnostic performance of EBUS-TBNA in lung cancer with non-enlarged lymph nodes has only been primarily studied in non-TB endemic country. Herth et al. reported a 89% to 92% sensitivity of EBUS-TBNA for determining the nodal status of NSCLC patients in whom the mediastinum appears normal on radiography [17], [18], a condition defined by lymph nodes with short-axis diameters <1 cm on contrast-enhanced CT. The current study conducted in a TB-endemic country also primarily aimed at lung cancer patients with non-enlarged lymph nodes. The result has shown an 80.6% sensitivity of EBUS-TBNA, which was comparable to the historical results in non-TB endemic area.. We reported a 53.8% negative predictive value of CT-defined negative mediastinal and hilar lymph nodes, and this value is not satisfactory but is similar to the result reported in another study [22]. Furthermore, our results show that PET has low diagnostic accuracy (47.7%) for determining nodal status, which can be mainly attributed to a high rate of false-positive results (46.2%), a negative diagnostic impact of PET frequently happened in TB-endemic areas.

The development of FDG-PET is considered major progress in the field of oncology that has led to advancement of assessment of the functional and molecular bases of tumors. However, F^18^-uptake in activated white blood cells interferes with that in tumor cells; therefore, false-positive findings in inflammatory and granulomatous lung diseases can be attributed to interference in F^18^-uptake [23]. Several reports from Asian countries indicate that PET has a 15–25% chance of overstaging potentially operable NSCLC [11], [12]. Management of these patients would be substantially altered accordingly if an invasive technique for nodal staging were unavailable [24], [25]. A history of pulmonary TB, despite inactive infection at the time of assessment, was reported to be the leading cause for the false-positive PET results [10], [11], [12], [26]. In our study, among the patients with lymph nodes yielding negative diagnostic impact of PET, 64.7% had various inflammatory lung diseases. Previous pulmonary TB was the leading cause of inflammatory lung disease, followed by pneumoconiosis. These parenchymal lung diseases develop from focal inflammation and spread via lymphatic drainage [27]. Hilar lymph nodes are the sentinel gates that prevent infectious or pro-inflammatory substances from reaching the mediastinum. This may be attributed to the significantly high rate of false-positive PET results for hilar lymph nodes. EBUS-TBNA could balance the weakness of PET in such condition. In our study, a negative EBUS-TBNA finding from a hilar node provided a 94.1% negative predictive value. On the other hand, a same finding from mediastinal lymph nodes showed an 80% negative predictive value. A large proportion of false-positive PET hilar lymph nodes in patients with no previous history of TB may be attributed to primary TB infection since a high percentage of adults in Taiwan are purified protein derivative (PPD)-positive [28], [29].

In our study, due to a high rate of false-positive results, the accuracy of PET for nodal diagnosis in cases of lung cancer patients with non-enlarged mediastinal and hilar lymph nodes did not increase. However, our results do not dispute the value of PET in detecting malignant lymph nodes on the basis of ^18^F-FDG uptake by the lymph nodes. The high sensitivity (85.7%) suggests that PET potentially can be used as a screening method to detect lymph nodes that are <1 cm. However, the high false-positive rate observed in hilar nodes warrants further investigation. A dual-time-point evaluation of FDG uptake, which combines standard uptake value with a retention index, has been proposed to improve lymph node diagnosis in NSCLC [30], [31]. However, other studies have reported controversial results of the superiority of dual-time-point evaluation technique over conventional VAS [32], [33]. On the other hand, our results show that the sensitivity and specificity of PET for distant metastasis is high; therefore, PET is an important staging tool to exclude distant metastasis.

In current study, we included lung cancer patients with non-enlarged lymph nodes to investigate the frequent clinical discrepancy between CT and PET results, but this was at the expense of a larger study population. However, the power of primary aim was not hampered by sample size because of the large effect size, i.e. the diagnostic accuracy difference of EBUS-TBNA and PET. In addition, PET imaging was performed on an older PET-only scanner, without CT correlation to intra-thoracic structures. This might be difficult for PET readers to determine whether the activity seen on PET correlates to lymph node or other structures. Whether use of integrated PET/CT might improve the accuracy deserves further investigation. However, given the dated technology of the scanner, it may not be enough to overcome the limited nature of PET arm of this study.

In conclusion, a high proportion of lung cancer patients with non-enlarged mediastinal and hilar lymph nodes on contrast-enhanced CT were found to have mediastinal or hilar lymph node metastasis. In a TB-endemic area, a high rate of negative diagnostic impact limits the clinical usefulness of PET for nodal staging; therefore, we recommend direct tissue sampling using EBUS-TBNA for nodal staging.

## References

[1] Doddoli C, Aragon A, Barlesi F, Chetaille B, Robitail S (2005). Does the extent of lymph node dissection influence outcome in patients with stage I non-small-cell lung cancer?. Eur J Cardiothorac Surg.

[2] Gajra A, Newman N, Gamble GP, Kohman LJ, Graziano SL (2003). Effect of number of lymph nodes sampled on outcome in patients with stage I non-small-cell lung cancer.. J Clin Oncol.

[3] Keller SM, Adak S, Wagner H, Johnson DH (2000). Mediastinal lymph node dissection improves survival in patients with stages II and IIIa non-small cell lung cancer. Eastern Cooperative Oncology Group.. Ann Thorac Surg.

[4] Kramer H, Groen HJ (2003). Current concepts in the mediastinal lymph node staging of nonsmall cell lung cancer.. Ann Surg.

[5] Guhlmann A, Storck M, Kotzerke J, Moog F, Sunder-Plassmann L (1997). Lymph node staging in non-small cell lung cancer: evaluation by [18F]FDG positron emission tomography (PET).. Thorax.

[6] Gupta NC, Graeber GM, Bishop HA (2000). Comparative efficacy of positron emission tomography with fluorodeoxyglucose in evaluation of small (<1 cm), intermediate (1 to 3 cm), and large (>3 cm) lymph node lesions.. Chest.

[7] Vansteenkiste JF, Stroobants SG, De Leyn PR, Dupont PJ, Verschakelen JA (1997). Mediastinal lymph node staging with FDG-PET scan in patients with potentially operable non-small cell lung cancer: a prospective analysis of 50 cases. Leuven Lung Cancer Group.. Chest.

[8] Bakheet SM, Powe J (1998). Benign causes of 18-FDG uptake on whole body imaging.. Semin Nucl Med.

[9] Roberts PF, Follette DM, von Haag D, Park JA, Valk PE (2000). Factors associated with false-positive staging of lung cancer by positron emission tomography.. Ann Thorac Surg.

[10] Kim YK, Lee KS, Kim BT, Choi JY, Kim H (2007). Mediastinal nodal staging of nonsmall cell lung cancer using integrated 18F-FDG PET/CT in a tuberculosis-endemic country: diagnostic efficacy in 674 patients.. Cancer.

[11] Turkmen C, Sonmezoglu K, Toker A, Yilmazbayhan D, Dilege S (2007). The additional value of FDG PET imaging for distinguishing N0 or N1 from N2 stage in preoperative staging of non-small cell lung cancer in region where the prevalence of inflammatory lung disease is high.. Clin Nucl Med.

[12] Konishi J, Yamazaki K, Tsukamoto E, Tamaki N, Onodera Y (2003). Mediastinal lymph node staging by FDG-PET in patients with non-small cell lung cancer: analysis of false-positive FDG-PET findings.. Respiration.

[13] Yasufuku K, Chiyo M, Sekine Y, Chhajed PN, Shibuya K (2004). Real-time endobronchial ultrasound-guided transbronchial needle aspiration of mediastinal and hilar lymph nodes.. Chest.

[14] Yasufuku K, Chiyo M, Koh E, Moriya Y, Iyoda A (2005). Endobronchial ultrasound guided transbronchial needle aspiration for staging of lung cancer.. Lung Cancer.

[15] Herth FJ, Eberhardt R, Vilmann P, Krasnik M, Ernst A (2006). Real-time endobronchial ultrasound guided transbronchial needle aspiration for sampling mediastinal lymph nodes.. Thorax.

[16] Herth FJ, Annema JT, Eberhardt R, Yasufuku K, Ernst A (2008). Endobronchial ultrasound with transbronchial needle aspiration for restaging the mediastinum in lung cancer.. J Clin Oncol.

[17] Herth FJ, Eberhardt R, Krasnik M, Ernst A (2008). Endobronchial ultrasound-guided transbronchial needle aspiration of lymph nodes in the radiologically and positron emission tomography-normal mediastinum in patients with lung cancer.. Chest.

[18] Herth FJ, Ernst A, Eberhardt R, Vilmann P, Dienemann H (2006). Endobronchial ultrasound-guided transbronchial needle aspiration of lymph nodes in the radiologically normal mediastinum.. Eur Respir J.

[19] Cerfolio RJ, Bryant AS, Eloubeidi MA, Frederick PA, Minnich DJ The true false negative rates of esophageal and endobronchial ultrasound in the staging of mediastinal lymph nodes in patients with non-small cell lung cancer.. Ann Thorac Surg.

[20] Lee HS, Lee GK, Lee HS, Kim MS, Lee JM (2008). Real-time endobronchial ultrasound-guided transbronchial needle aspiration in mediastinal staging of non-small cell lung cancer: how many aspirations per target lymph node station?. Chest.

[21] Wallace MB, Pascual JM, Raimondo M, Woodward TA, McComb BL (2008). Minimally invasive endoscopic staging of suspected lung cancer.. Jama.

[22] Takamochi K, Nagai K, Yoshida J, Suzuki K, Ohde Y (2000). The role of computed tomographic scanning in diagnosing mediastinal node involvement in non-small cell lung cancer.. J Thorac Cardiovasc Surg.

[23] Castell F, Cook GJ (2008). Quantitative techniques in 18FDG PET scanning in oncology.. Br J Cancer.

[24] Pieterman RM, van Putten JW, Meuzelaar JJ, Mooyaart EL, Vaalburg W (2000). Preoperative staging of non-small-cell lung cancer with positron-emission tomography.. N Engl J Med.

[25] Schrevens L, Lorent N, Dooms C, Vansteenkiste J (2004). The role of PET scan in diagnosis, staging, and management of non-small cell lung cancer.. Oncologist.

[26] Takamochi K, Yoshida J, Murakami K, Niho S, Ishii G (2005). Pitfalls in lymph node staging with positron emission tomography in non-small cell lung cancer patients.. Lung Cancer.

[27] Gawne-Cain ML, Hansell DM (1996). The pattern and distribution of calcified mediastinal lymph nodes in sarcoidosis and tuberculosis: a CT study.. Clin Radiol.

[28] Bowerman RJ (2004). Tuberculin skin testing in BCG-vaccinated populations of adults and children at high risk for tuberculosis in Taiwan.. Int J Tuberc Lung Dis.

[29] Yeh YP, Luh DL, Chang SH, Suo J, Chang HJ (2005). Tuberculin reactivity in adults after 50 years of universal bacille Calmette-Guerin vaccination in Taiwan.. Trans R Soc Trop Med Hyg.

[30] Kubota K, Itoh M, Ozaki K, Ono S, Tashiro M (2001). Advantage of delayed whole-body FDG-PET imaging for tumour detection.. Eur J Nucl Med.

[31] Matthies A, Hickeson M, Cuchiara A, Alavi A (2002). Dual time point 18F-FDG PET for the evaluation of pulmonary nodules.. J Nucl Med.

[32] Nishiyama Y, Yamamoto Y, Kimura N, Ishikawa S, Sasakawa Y (2008). Dual-time-point FDG-PET for evaluation of lymph node metastasis in patients with non-small-cell lung cancer.. Ann Nucl Med.

[33] Yen RF, Chen KC, Lee JM, Chang YC, Wang J (2008). 18F-FDG PET for the lymph node staging of non-small cell lung cancer in a tuberculosis-endemic country: is dual time point imaging worth the effort?. Eur J Nucl Med Mol Imaging.

